# The Fat Mass and Obesity Associated Gene *FTO* Functions in the Brain to Regulate Postnatal Growth in Mice

**DOI:** 10.1371/journal.pone.0014005

**Published:** 2010-11-16

**Authors:** Xue Gao, Yong-Hyun Shin, Min Li, Fei Wang, Qiang Tong, Pumin Zhang

**Affiliations:** 1 Department of Biochemistry and Molecular Biology, Baylor College of Medicine, Houston, Texas, United States of America; 2 Department of Molecular Physiology and Biophysics, Baylor College of Medicine, Houston, Texas, United States of America; 3 Department of Pediatrics, Children's Nutrition Research Center, Baylor College of Medicine, Houston, Texas, United States of America; Louisiana State University, United States of America

## Abstract

*FTO* (fat mass and obesity associated) was identified as an obesity-susceptibility gene by several independent large-scale genome association studies. A cluster of SNPs (single nucleotide polymorphism) located in the first intron of *FTO* was found to be significantly associated with obesity-related traits, such as body mass index, hip circumference, and body weight. *FTO* encodes a protein with a novel C-terminal α-helical domain and an N-terminal double-strand β-helix domain which is conserved in Fe(II) and 2-oxoglutarate-dependent oxygenase family. *In vitro*, FTO protein can demethylate single-stranded DNA or RNA with a preference for 3-methylthymine or 3-methyluracil. Its physiological substrates and function, however, remain to be defined. Here we report the generation and analysis of mice carrying a conditional deletion allele of *Fto*. Our results demonstrate that *Fto* plays an essential role in postnatal growth. The mice lacking *Fto* completely display immediate postnatal growth retardation with shorter body length, lower body weight, and lower bone mineral density than control mice, but their body compositions are relatively normal. Consistent with the growth retardation, the *Fto* mutant mice have reduced serum levels of IGF-1. Moreover, despite the ubiquitous expression of *Fto*, its specific deletion in the nervous system results in similar phenotypes as the whole body deletion, indicating that *Fto* functions in the central nerve system to regulate postnatal growth.

## Introduction

The mouse gene *Fto* was first cloned more than a decade ago as one of the several genes deleted in the Fused toes (*Ft*) mutant mouse created by insertional mutagenesis [1]. However, it did not draw much attention until very recently when its human homolog *FTO* was implicated in obesity. In 2007, several groups reported that a cluster of SNPs (single nucleotide polymorphism) in the first intron of *FTO* was highly associated with obesity-related traits and higher obesity risk [2]–[4]. The association has been further confirmed by other independent studies in different human populations [5]–[15], thus rendering *FTO* the most likely culprit for common forms of obesity. Subsequently, several studies suggested those obesity-associated SNPs were correlated with higher energy intake, increased appetite [16]–[21] without affecting energy expenditure [18]–[20], [22] or physical activity [17], [22]–[24], although one study also reported no association with energy intake [24]. However, since the obesity-associated SNPs do not affect the coding region of *FTO*, whether the connection to obesity works through the function of *FTO* per se is still a question, since the SNPs may exert influence on the expression of distant genes other than *FTO*.

The FTO protein contains a double-stranded β-helix fold typical of the members in the Fe(II) and 2-oxoglutarate-dependent oxygenase family [25]–[27]. This enzyme family has diverse biological functions all based on the similar chemical mechanism [28], [29]. Indeed, similar to a number of members in this family, FTO can demethylate single-stranded nucleic acids *in vitro* [25], [26], [30]. However, the physiological function and *in vivo* substrates of FTO are not well defined.

Two *Fto* mutant mouse models have been reported before [31], [32]. The complete knockout mice displayed growth retardation and reduced adiposity [31]. The other mice model bearing a missense mutation in *Fto* developed a lean phenotype later in life while the linear growth remained unaffected [32]. The studies so far seemed to connect the deficiency of FTO with protection of obesity in mice.

Here we described the generation and characterization of two mouse models with varying *Fto* deficiencies. The whole body *Fto* knockout mice displayed immediate postnatal growth retardation, with shorter body length, lower body weight, and lower bone mineral density than control mice. However, the mutant mice had relatively normal body composition and were still susceptible to diet induced obesity. In another mouse model, *Fto* was specifically deleted in the neural system. Despite the ubiquitous expression of *Fto*, its specific deletion in the nervous system results in similar growth retardation phenotypes as the whole body deletion, suggesting that *Fto* functions in the brain to regulate postnatal growth.

## Results

### FTO protein expression in mice

**Figure 1:**
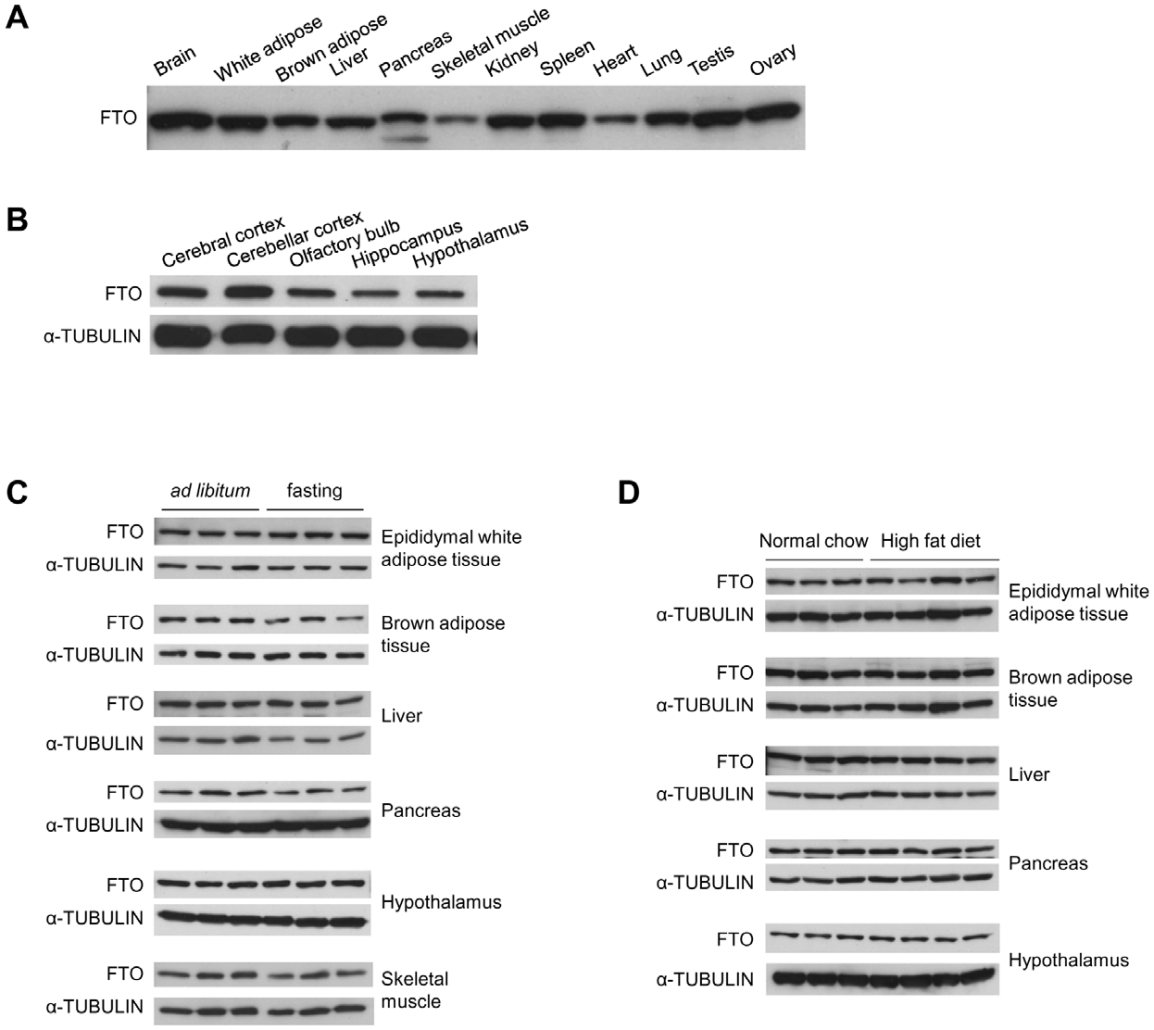
FTO protein is ubiquitously expressed in mouse tissues and not affected by nutritional status in mice. (**A**) Tissues from adult male C57BL/6 mice (except the ovary) were homogenized and immunoblotted. 20 µg total protein was loaded in each lane. (**B**) Expression of FTO in different regions of the brain. (**C**) Western blot analysis of FTO in metabolism-related tissues from adult male C57BL/6 mice fed *ad libitum* or fasted for 24 hrs. (**D**) Western blot analysis of FTO in tissues from C57BL/6 male mice fed on normal chow or on high fat diet (60 kcal % fat) from 6-week-old for 17 weeks.

We raised antibodies in rabbit against full-length mouse FTO protein and surveyed the expression in various mouse tissues with immunoblotting. Consistent with the previous RT-PCR results [26], the expression of FTO protein was detected in all of the major mouse tissues examined, with the highest level of expression in the brain and the lowest in the skeletal muscle (Figure 1A). Within the brain, FTO is expressed more or less uniformly in different anatomical structures, such as hypothalamus and hippocampus (Figure 1B). Given the association of *FTO* with human obesity, we wished to determine if the expression of FTO is influenced by food intake. Adult male C57BL/6 mice were fasted for 24 hrs and the FTO expression was examined in energy metabolism-related tissues including white adipose tissue, brown adipose tissue, liver, pancreas, hypothalamus, and skeletal muscle (Figure 1C). However, no obvious changes in the expression of FTO were detected. We also fed male C57BL/6 mice with high fat diet for 17 weeks starting from 6-week-old, and the FTO protein level did not show noticeable changes either (Figure 1D). Thus, if *Fto* is regulated by food status or type, the regulation is unlikely at the level of protein expression. Previous studies have shown that the *Fto* mRNA level in the arcuate nucleus (ARC) of the hypothalamus is reduced by fasting in mice [26], and increased by exposure to high fat diet in rats [33]. It is possible these regional changes cannot be detected in the Western blot of whole hypothalamus.

### Generation of *Fto* deletion mice

**Figure 2:**
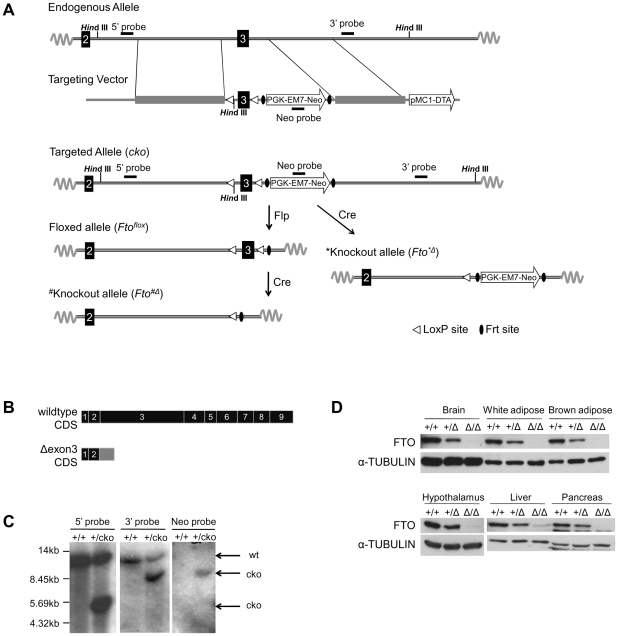
Generation of *Fto* knockout mice. (**A**) Strategy to generate various *Fto* alleles. Hin*d III* indicates the enzyme cutting sites for Southern blot analysis in (C). (**B**) Schematic representation of predicted *Fto* wildtype and knockout coding sequences (CDS). (**C**) Southern blot analysis of wildtype and *cko* alleles. The genomic DNA was digested by *Hin*d III, separated on agarose gel, blotted, and hybridized with the indicated probes as shown in (A). (**D**) Western blot analysis of different tissues isolated from wildtype (+/+), heterozygous (+/Δ) and homozygous (Δ/Δ) mice.

In order to investigate the physiological function of *Fto* in mice, we generated a conditional knockout (cko) line by flanking exon 3 with two loxP sites (Figure 2A). Exon 3 encodes about 40% of the protein. Deletion of exon 3 would result in frame-shift of the downstream exons and early termination in translation (Figure 2B). Germline transmission of the cko allele (*Fto^cko^*) was obtained and confirmed by Southern blot analysis (Figure 2C). F1 *Fto^+/cko^* mice were either directly bred to Meox2-Cre [34] mice to generate a knockout allele (Fto^*Δ^) still bearing the neo selection cassette, or first bred to Flpase-expressing mice [35] to generate a floxed allele (*Fto^flox^*). *Fto^+/flox^* mice were then crossed to Meox2-Cre mice to generate the clean deletion of exon 3 allele (*Fto^#Δ^*) (Figure 2A). Both *Fto^#Δ^* and Fto^*Δ^ were used as knockout in this study and will be referred together as *Fto^Δ^* from hereon, since there is no phenotypic difference between Fto^#Δ/#Δ^ and Fto^*Δ/*Δ^ mice. Homozygous knockout mice (*Fto^Δ/Δ^*) were obtained by crossing of heterozygous pairs, and the absence of FTO protein expression in *Fto^Δ/Δ^* mice was confirmed by Western blot analysis (Figure 2D).

### Complete depletion of *Fto* in mice results in postnatal growth retardation

**Figure 3:**
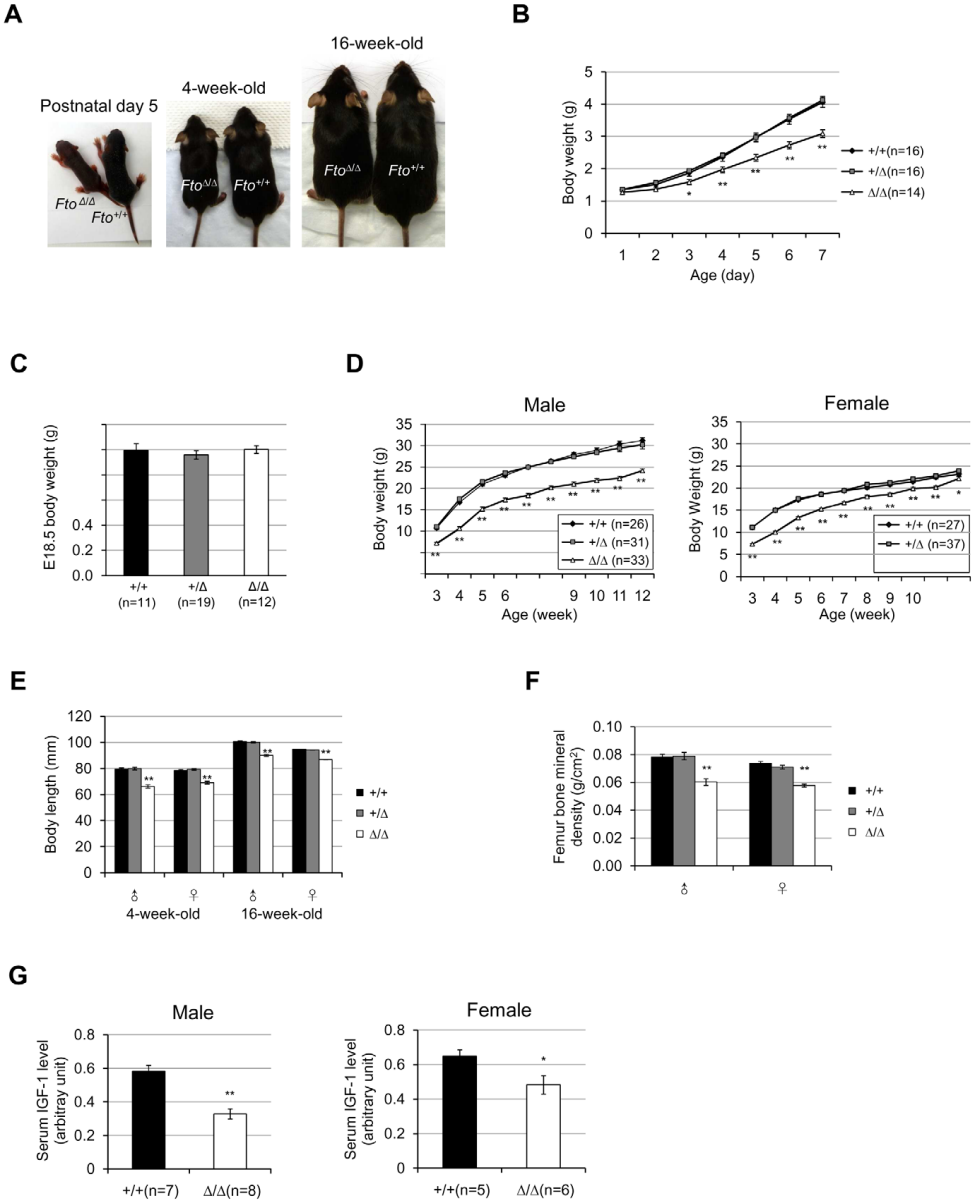
Complete knockout of *Fto* results in postnatal growth retardation. (**A**) Representative pictures of wildtype and *Fto* knockout mice at different ages. (**B**) Growth curves of *Fto^+/+^*, *Fto^+/Δ^*, and *Fto^Δ/Δ^* mice from postnatal day 1 to 7. n = 16/16/14 (*Fto^+/+^*/*Fto^+/Δ^*/*Fto^Δ/Δ^*). *P*-values from day 1 to 7: 0.29, 0.062, 0.011, 0.0069, 0.0007, <0.0001, and <0.0001. (**C**) Body weights of *Fto^+/+^*, *Fto^+/Δ^* and *Fto^Δ/Δ^* embryos at E18.5. n = 11/19/12 (*Fto^+/+^*/*Fto^+/Δ^*/*Fto^Δ/Δ^*). *P* = 0.68. (**D**) Growth curves of male and female *Fto^+/+^*, *Fto^+/Δ^* and *Fto^Δ/Δ^* mice. For each genotype (*Fto^+/+^*/*Fto^+/Δ^*/*Fto^Δ/Δ^*), n = 26/31/33 (male), n = 27/37/49 (female). **P<0.01 for all the time points, except in females at 12 weeks old, *P* = 0.024. (**E**) Body length of adolescent and adult *Fto^+/+^*, *Fto^+/Δ^* and *Fto^Δ/Δ^* mice. For each genotype (*Fto^+/+^*/*Fto^+/Δ^*/*Fto^Δ/Δ^*), n = 12/16/17 (4-week-old male), and 11/13/15 (4-week-old female); n = 14/13/13 (16-week-old male), and 17/25/32 (16-week-old female). (**F**) Bone mineral density of 16-week-old *Fto^+/+^*, *Fto^+/Δ^* and *Fto^Δ/Δ^* mice. The femur bone mineral densities were measured by DEXA (dual energy X-ray absorptiometry). For each genotype (*Fto^+/+^*/*Fto^+/Δ^*/*Fto^Δ/Δ^*), n = 14/13/13 (male); n = 17/25/32 (female). (**G**) Relative serum IGF-1 levels of 4-week-old mice. The serum IGF-1 levels were measured with ELISA. For each genotype (*Fto^+/+^*/*Fto^Δ/Δ^*), n = 7/8 (male), n = 5/6 (female). *P*<0.0001 (male), *P* = 0.0356 (female). Statistical analyses were performed by one-way ANOVA, except that unpaired t-test was used in (G). *P<0.05, **P<0.01. All values are mean ± s.e.m.

*Fto^Δ/Δ^* mice are viable, but only about 50% of them could survive to postnatal day 10–14 (Table S1) as has been reported in another *Fto* knockout mouse model [31], [36]. The death mostly happened within a few days after birth (Table S2). It is unclear what causes the death at present. However, near term *Fto^Δ/Δ^* embryos were recovered at the Mendelian ratio (Table S3), indicating that *Fto* is not required for embryogenesis in mice and the death mostly likely happened after the birth. Surviving *Fto^Δ/Δ^* mice displayed immediate growth retardation in both males and females (Figure 3A). Within the first few days after the birth, the *Fto^Δ/Δ^* mice were already significantly lighter in weight comparing to their wildtype and heterozygous littermates, and the difference increased over time (Figure 3B). This is unlikely a result of that the mutant pups were born smaller than normal, because there was no weight difference among embryos of all three genotypes at E18.5 (Figure 3C). At the time of weaning, both male and female *Fto^Δ/Δ^* mice were about 65% the weight of wildtype and heterozygous littermates (Figure 3D). While the male *Fto^Δ/Δ^* mice remained behind throughout adulthood, the females showed a trend of catching up in weight later on (Figure 3D). *Fto^Δ/Δ^* mice were also significantly shorter in body length throughout lifetime (Figure 3E), and they had much lower bone mineral density (Figure 3F). Consistent with the growth retardation phenotype, *Fto^Δ/Δ^* mice had significantly lower serum levels of IGF-1 (insulin-like growth factor 1) than the controls at the time of growth spurt (Figure 3G).

### *Fto^Δ/Δ^* mice do not display a lean phenotype

**Figure 4:**
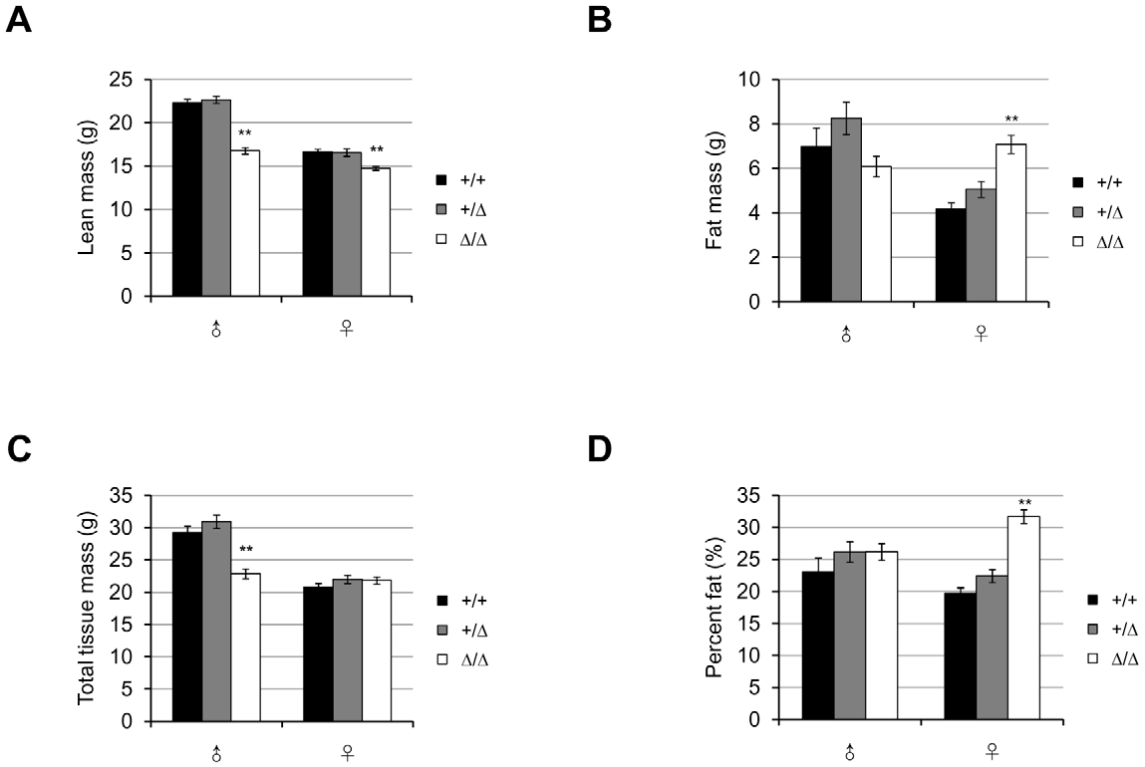
*Fto* knockout mice do not display a lean phenotype. (**A**, **B**, **C**) Lean mass (A), fat mass (B) and total tissue mass (C) of 16-week-old mice of each genotype. (**D**) Body composition (fat mass/total tissue mass %) of 16-week-old mice of each genotype. *P* = 0.35(male), and *P*<0.0001(female). In (A)–(D), all mice were fed on normal chow after weaning at 3-week-old and were 16-week-old at the time of DEXA (dual energy X-ray absorptiometry) measurement. For each genotype (*Fto^+/+^*/*Fto^+/Δ^*/*Fto^Δ/Δ^*), n = 14/13/13 (male), 17/25/32 (female). Statistical analyses were performed by one-way ANOVA. **P<0.01. All values are mean ± s.e.m.

To determine whether *Fto*-deficiency affects body fat mass, the body compositions of 16-week-old mice fed on normal chow were measured by DEXA (dual energy X-ray absorptiometry). Despite the general decrease in size, *Fto^Δ/Δ^* mice did not show a specific reduction in fat. Among the 16-week-old males, *Fto^Δ/Δ^* mice had about 25% less lean mass than *Fto^+/+^* or *Fto^+/Δ^* mice (Figure 4A) and slightly less fat mass (statistically not significant) (Figure 4B). When normalized to the total tissue weight (lean mass plus fat mass) (Figure 4C), male mice of all three genotypes had similar fat content (fat mass/total tissue mass %) (Figure 4D). On the other hand, at the age of 16 weeks, female *Fto^Δ/Δ^* mice had even more fat mass on average than their wildtype and heterozygous littermates (Figure 4B), while their lean mass were still less than the controls (about 12% less, not as dramatic as the difference in males) (Figure 4A). As a result, female *Fto^Δ/Δ^* mice contained higher proportion of fat (fat mass/total tissue mass %) than wildtype or heterozygous mice (Figure 4D). The increased fat mass in mutant females compensated for their slight deficit in lean mass, and made it the total tissue mass even (Figure 4C), which also explains the trend of catching-up in whole body weight as the mice got older (Figure 3D).

### *Fto^Δ/Δ^* mice are susceptible to diet-induced-obesity

**Figure 5:**
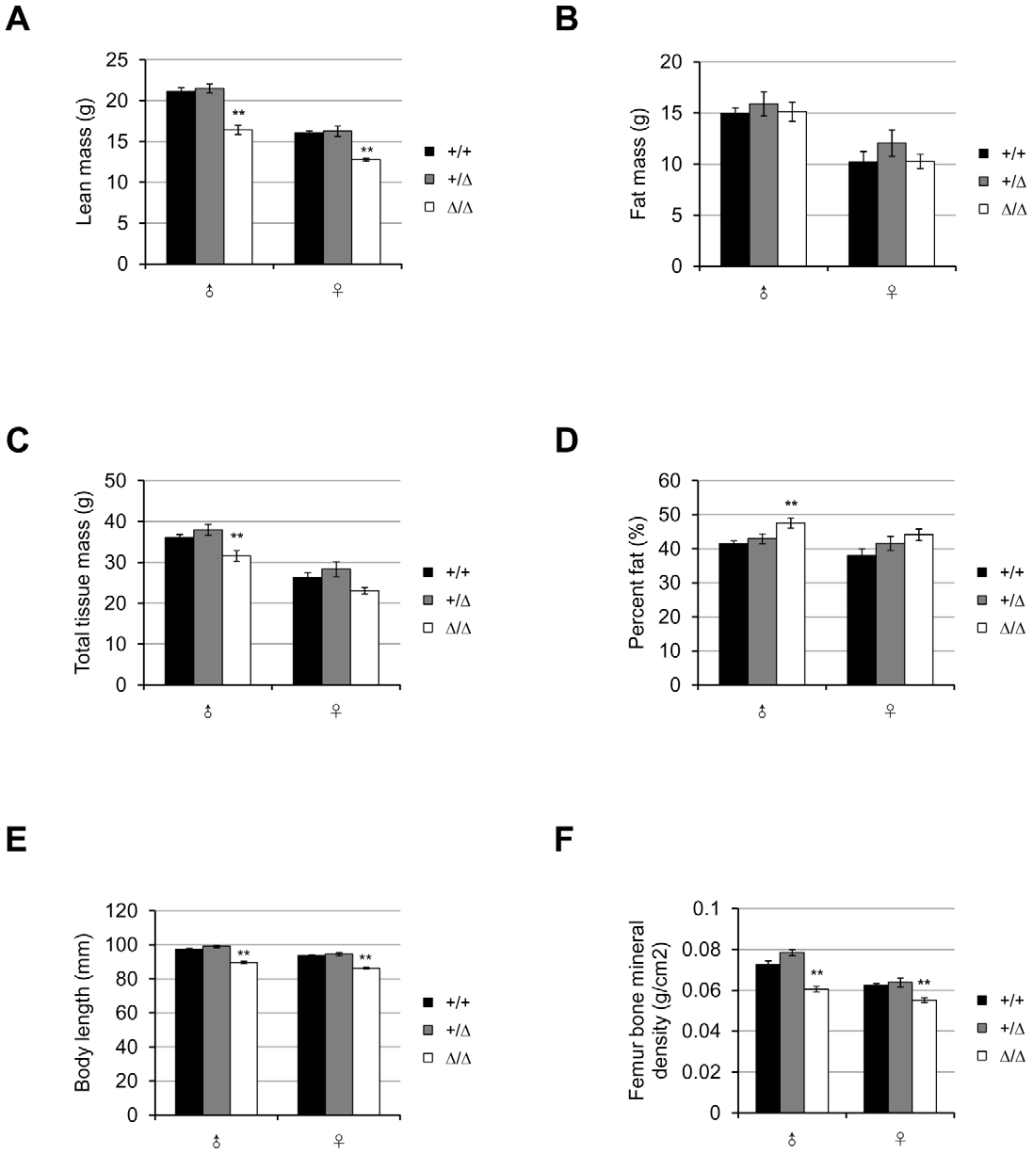
*Fto^Δ/Δ^* mice are susceptible to high fat diet-induced obesity. (**A**, **B**, **C**) Lean mass (A), fat mass (B) and total tissue mass (C) of *Fto^+/+^*, *Fto^+/Δ^* and *Fto^Δ/Δ^* mice on high fat diet. (**D**) Body composition (fat mass/total tissue mass %) of *Fto^+/+^*, *Fto^+/Δ^* and *Fto^Δ/Δ^* mice on high fat diet. *P* = 0.0055 (male), *P* = 0.1(female). (**E**, **F**) Body length (E) and femur bone mineral density (F) of *Fto^+/+^*, *Fto^+/Δ^* and *Fto^Δ/Δ^* mice on high fat diet. In (A)–(F), all mice were fed on 60 kcal% fat diet for 10 weeks starting from 4-week-old. DEXA measurements were performed at the end of the 10-week period. For each genotype (*Fto^+/+^*/*Fto^+/Δ^*/*Fto^Δ/Δ^*), n = 14/14/12 (male), 11/9/8 (female). Statistical analyses were performed by one-way ANOVA. **P<0.01. All values are mean ± s.e.m.

To determine if the status of *Fto* has an effect on the response to diet, both male and female mice of three genotypes (*Fto^+/+^*, *Fto^+/Δ^* and *Fto^Δ/Δ^*) were fed on high fat diet (60 kcal % fat) from 4-week-old on for 10 weeks. Unexpectedly, about half of the *Fto^Δ/Δ^* mice on high fat diet developed dermatitis (Figure S2A, Table S4) at the late stage of the diet regimen, and they became very lean due to the illness (Figure S2B). The reason of this illness is unclear. The dermatitis-free *Fto^Δ/Δ^* mice did respond to high fat diet and developed DIO (diet-induced-obesity) (Figure S2B). At the end of the diet regimen, all the mice free of dermatitis were measured by DEXA (dual energy X-ray absorptiometry). The *Fto^Δ/Δ^* mice still had significantly less lean mass than wildtype and heterozygotes (Figure 5A), while they had the similar amount of fat mass (Figure 5B). When normalized to total tissue mass (Figure 5C), the *Fto^Δ/Δ^* mice had relatively higher percentage of fat mass than controls, though not statistically significant in females (Figure 5D). At the same time, the *Fto^Δ/Δ^* mice on high fat diet retained other aspects of the growth retardation phenotype, such as shorter body length (Figure 5E) and lower bone mineral density (Figure 5F).

### *Fto^Δ/Δ^* mice have higher metabolic rates

**Figure 6:**
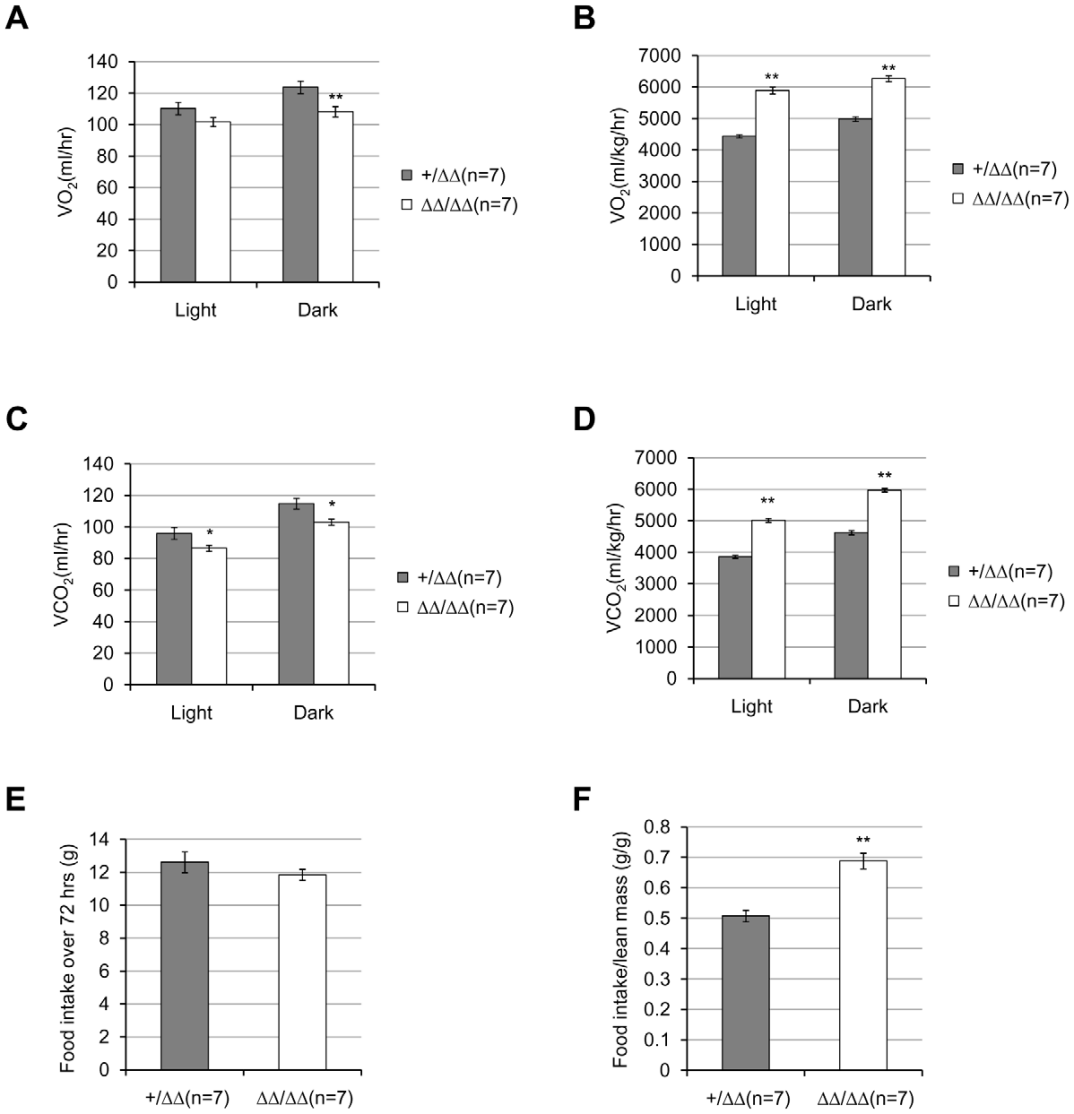
Metabolic parameters in *Fto* complete knockout mice. (**A**) Average hourly oxygen consumption of 16∼17-week-old male *Fto^+/Δ^* and *Fto^Δ/Δ^* mice during the light and dark period. *P* = 0.1044 (light), and *P* = 0.0098(dark). (**B**) Average hourly oxygen consumption divided by lean mass. *P*<0.0001. (**C**) Average hourly carbon dioxide production of 16∼17-week-old male *Fto^+/Δ^* and *Fto^Δ/Δ^* mice during the light and dark period. *P* = 0.0463 (light), and *P* = 0.011(dark). (**D**) Average hourly carbon dioxide production divided by lean mass. *P*<0.0001. (**E**) Accumulative food intake over a period of 72 hours of 16∼17-week-old male *Fto^+/Δ^* and *Fto^Δ/Δ^* mice. *P* = 0.3015. (**F**) Food intake divided by lean mass. *P* = 0.0001. The number of mice used was 7 for each genotype. Statistical analyses were performed by unpaired t-test. **P<0.01. All values are mean ± s.e.m.

To determine whether the deletion of *Fto* would affect the metabolism in mice, the O_2_ consumption and CO_2_ production of 16∼17-week-old male mice were measured using indirect caloriometry. As there was no phenotypic difference in wildtype and heterozygous mice, only *Fto^+/Δ^* and *Fto^Δ/Δ^* mice were used here due to the availability of mice at the time of experiment. When the absolute amount of O_2_ consumption (VO_2_) and CO_2_ production (VCO_2_) were measured, *Fto^Δ/Δ^* mice showed relatively lower rates of O_2_ consumption and CO_2_ production than *Fto^+/Δ^* mice (Figure 6A, C), during both light and dark periods (though the difference of VO_2_ during light period was not statistically significant). When divided by lean mass (conventional normalization), the *Fto^Δ/Δ^* mice displayed significantly higher metabolic rates (Figure 6B, D), possibly due to their much-reduced lean mass. When the data was analyzed by ANCOVA using lean mass as a covariant, the *Fto^Δ/Δ^* mice still showed higher O_2_ consumption and CO_2_ production rates during both light and dark period (Figure S3A–D), suggesting the deletion of *Fto* increases the metabolic rate, even after its effect on body mass has been taken into account.

While the oxygen consumption was measured, we also measured the food intake of *Fto* mutant mice. In a period of 72 hours, *Fto^+/Δ^* and *Fto^Δ/Δ^* mice consumed similar amount of food (Figure 6E). However, *Fto^Δ/Δ^* mice appeared to have consumed more food when the amount of food intake was normalized to lean mass (Figure 6F).

Taken together, these data suggest that *Fto* affects the food intake and energy expenditure at the same time. In its absence, both are increased.

### Neural-specific *Fto* knockout mice are phenotypically similar to the complete knockout

**Figure 7:**
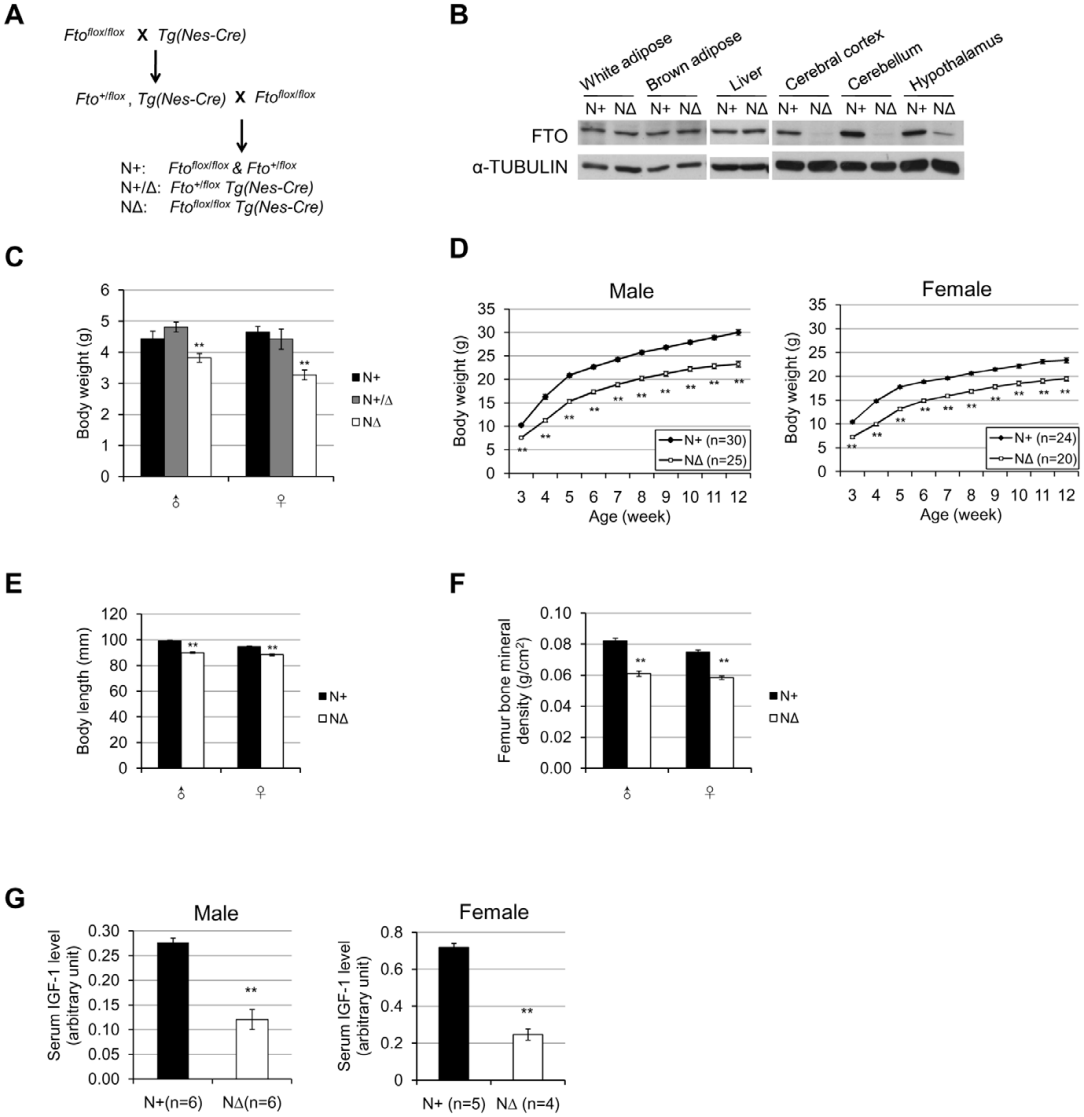
Neural-specific *Fto* knockout mice are growth retarded. (**A**) The breeding scheme to generate neural-specific *Fto* knockout mice. (**B**) Western blot analysis of different tissues of *Fto^N+^* and *Fto^NΔ^* mice. (**C**) Body weights of 7-day-old *Fto^N+/+^*, *Fto^N+/Δ^* and *Fto^NΔ/Δ^* pups. For each genotype (*Fto^N+/+^*/*Fto^N+/Δ^*/*Fto^NΔ/Δ^*), n = 17/7/9 (male), and 14/7/8 (female). (**D**) Growth curves of male and female *Fto^N+^* and *Fto^NΔ^* mice. For each genotype (*Fto^N+^*/*Fto^NΔ^*), n = 30/25(male), and 24/20(female). (**E**, **F**) Body length (E) and femur bone mineral density (F) of 16-week-old *Fto^N+^* and *Fto^NΔ^* mice measured by DEXA. For each genotype (*Fto^N+^*/*Fto^NΔ^*), n = 19/21(male), 19/17(female). (**G**) Relative serum IGF-1 levels of 4-week-old *Fto^N+^* and *Fto^NΔ^* mice. For each genotype (*Fto^N+^*/*Fto^NΔ^*), n = 6/6(male), and n = 5/4(female). Statistical analyses were performed by one-way ANOVA (C) or unpaired t-test (D–G). **P<0.01. All values are mean ± s.e.m.

Before we generated the *Fto* conditional knockout line, we had made another genetrap mouse line (Figure S1A, B) which still had residual expression of wildtype FTO (Figure S1C). It is interesting that homozygous genetrap mice (*Fto^gt/gt^*) displayed none of the phenotypes (Figure S1D–F) of *Fto^Δ/Δ^* mice. It is possible the residual FTO in the peripheral tissues may be enough for its function, considering *Fto* heterozygous knockout mice, as well as humans heterozygous for the *FTO* loss of function mutations are generally normal [37]. On the other hand, since there was only minimal reduction of FTO in the brain tissues of the gene-trap mice comparing to other tissues (Figure S1C), we reasoned that *Fto* might function in the brain, To explore this possibility, we deleted *Fto* in the nervous system by crossing *Fto^flox^* allele with the Nestin-Cre transgenic mice [38] (Figure 7A). Western blotting analysis of *Fto^flox/flox^/Nestin-Cre* (will be referred to as *Fto^NΔ^* hereafter, *N* denotes neural) mice confirmed the efficient reduction of FTO protein in the brain, but not the peripheral tissues (Figure 7B). These mice displayed growth retardation, similar to the complete knockout mice (Figure 7C, D). Both male and female *Fto^NΔ^* mice had shorter body length (Figure 7E) and lower bone mineral density (Figure 7F). *Fto^NΔ^* mice also had much lower serum levels of IGF-1 during growth spurt (Figure 7G).

### Neural-specific deletion of *Fto* affects body composition of mice in a similar way as the whole body knockout

**Figure 8:**
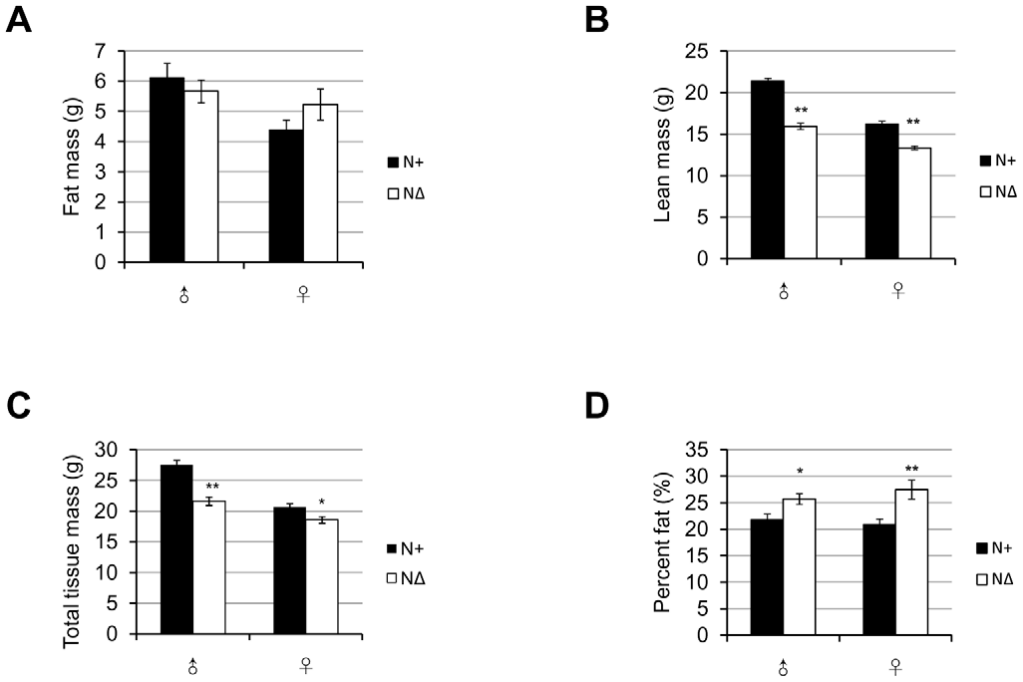
The body composition of *Fto* neural-specific knockout mice. (**A**) Fat mass of 16-week-old *Fto^N+^* and *Fto^NΔ^* mice. *P* = 0.4551(male), 0.1681(female). (**B**) Lean mass of 16-week-old *Fto^N+^* and *Fto^NΔ^* mice. (**C**) Total tissue mass of 16-week-old *Fto^N+^* and *Fto^NΔ^* mice. *P*<0.0001(male), and *P* = 0.0175(female). (**D**) Body composition (fat mass/total tissue mass %) of 16-week-old *Fto^N+^* and *Fto^NΔ^* mice. *P* = 0.0118(male), 0.0024(female). In (A)–(D), for each genotype (*Fto^N+^*/*Fto^NΔ^*), n = 19/21(male), 19/17(female). Statistical analyses were performed by unpaired t-test. *P<0.05, **P<0.01. All values are mean ± s.e.m.

Similar to the whole body knockout, the body composition of 16-week old *Fto^NΔ^* mice didn't show any deficits in fat accumulation. The *Fto^NΔ^* mice had similar fat mass as to the controls, with a trend of slightly less fat mass in males, and more in females (Figure 8A). Here the difference in the fat mass in females was not statistically significant as in the complete knockout (possibly due to the incomplete depletion of *Fto*, the genetic background introduced by the *Nestin-Cre* transgene, or a sampling issue of individual variance). The deficit in lean mass of *Fto^NΔ^* mice remained the same as the complete knockout (Figure 8B). Taken together, the *Fto^NΔ^* mice had less total tissue mass than the controls (Figure 8C). When normalized to the total tissue mass, *Fto^NΔ^* mice had relatively higher fat content (fat mass/total tissue mass %) (Figure 8D), which was more obvious in the case of females, similar to the complete knockout mice (Figure 4D). Thus, the general trend of body composition (increasing fat content) in neural-specific *Fto* knockout is similar to that in the complete knockout.

### The metabolic parameters of neural-specific *Fto* knockout mice are increased similarly as in the complete knockout mice

**Figure 9:**
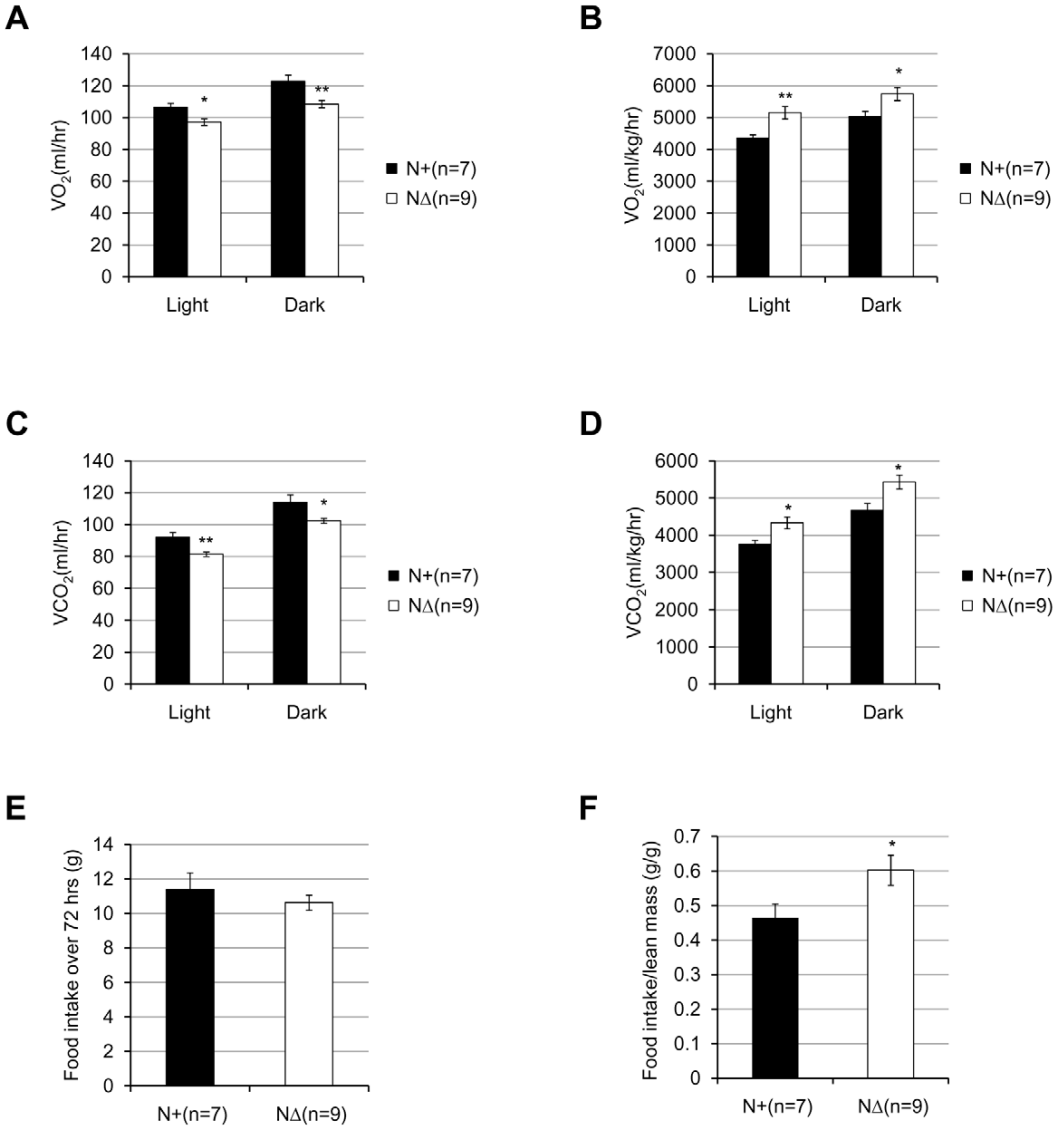
The metabolic parameters of *Fto* neural-specific knockout mice. (**A**) Average hourly oxygen consumption of 16∼17-week-old male *Fto^N+^* and *Fto^NΔ^* mice during the light and dark period. *P* = 0.0105 (light), and *P* = 0.0044 (dark). (**B**) Average hourly oxygen consumption divided by lean mass. *P* = 0.00556 (light), and *P* = 0.0233(dark). (**C**) Average hourly carbon dioxide production of 16∼17-week-old male *Fto^N+^* and *Fto^NΔ^* mice during the light and dark period. *P* = 0.004 (light), and *P* = 0.0196(dark). (**D**) Average hourly carbon dioxide production divided by lean mass. *P* = 0.0131(light), and *P* = 0.0142(dark). (**E**) Accumulative food intake over a period of 72 hours of 16∼17-week-old male *Fto^N+^* and *Fto^NΔ^* mice. *P* = 0.4601. (**F**) Food intake divided by lean mass. In (A)–(F), the number of animals used were 7 (*Fto^N+^*) and 9 (*Fto^NΔ^*). Statistical analyses were performed by unpaired t-test. *P<0.05, **P<0.01. All values are mean ± s.e.m.

The metabolic rates and food intake were also measured in 16∼17-week-old male *Fto^N+^* and *Fto^NΔ^* mice. Similarly, when the raw VO_2_ and VCO_2_ values were compared, *Fto^NΔ^* mice showed relatively lower O_2_ consumption and CO_2_ production rate than the control mice (Figure 9A, C), during both light and dark periods. Again, when normalized to lean mass by the simple division, the *Fto^NΔ^* mice seemed to have relatively higher metabolic rates (Figure 9B, D). As the set of data of neural-specific knockout mice did not show a significant linear relationship to lean mass within groups (Figure S3E, F), ANCOVA analyses could not be applied here.

*Fto^NΔ^* mice also had similar food consumption as of *Fto^N+^* mice during a period of 72 hours (Figure 9E), and appeared to have consumed more food when normalized to the lean mass (Figure 9F).

## Discussion

We report here the analysis of two mouse models of *Fto* deficiencies. The whole body deletion of *Fto* resulted in postnatal growth retardation manifested as reduced body weight and length, lower bone mineral density and lower serum IGF-1 levels, but did not affect fat accumulation when fed on either normal or high fat diet. Interestingly, the deletion of *Fto* in the nervous system resulted in the similar growth retardation phenotypes as the whole body knockout, indicating that *Fto* functions in the brain to regulate growth.

The strong growth retardation phenotype in our *Fto* deletion mice is consistent with that in the previously reported knockout mouse model generated by a slightly different strategy [31]. However, we did not observe a strong lean phenotype in our *Fto*-deficient mice as reported previously [32], [36]. Instead, we detected a trend of increasing fat mass content (fat mass/total tissue mass %) in the mutants when the mice were fed on normal chow, especially in the females. Both male and female knockout mice also responded well to high fat diet and developed obesity. The male mutants were even more susceptible to diet-induced-obesity than the controls. We did recognize that the individual variation in body fat mass was relatively large, even in the control groups, while the lean mass was quite constant among individuals. This variation might have come from both genetic and environmental factors and likely underlie the difference with the previous reports. Still, the absence of the specific lean phenotype and the lack of resistance to diet-induced-obesity in our *Fto* knockout mice are in contrast with the previous reports.

In the initial report of *Fto*-deficient mice, the mutant mice were stated to have increased enrgy expenditure when compared to controls, which was cited as the reason for the reduction in adiposity in spite of the relative hyperpahgia [31]. This explanantion was recently challenged based on the lack of statistic power and the oversimplification of normalization method [39], [40]. We also measured the metabolic rate (in terms of O_2_ consumption and CO_2_ production) using indirect caloriometry. While the unnormalized O_2_ consumption and CO_2_ production rates were decreased in *Fto* knockout mice, the correction for lean mass using either direct division or ANCOVA reversed the result, pointing to an increase of metabolic rates in the mutant mice. On the other hand, both ours and the previous study all indicated an increase in food intake caused by *Fto* deficiency [31]. A more recent report demosntrated that manipulating the FTO levels in the arcuate nucleus of hypothalamus in rats could affect the food intake [33]. Thus, it appears that the status of *Fto* has an impact on both the energy expenditure and food intake. In the absence of *Fto*, both of the two parameters are increased. Given the trend of more fat accumulation in our *Fto* knockout mice, the increase in the energy expenditure seemed overwhelmed by the increase in food intake.

A number of human studies suggested that the risk SNPs in *FTO* associate with increases in food intake [16]–[21], and rejected an effect on energy expenditure [18]–[20], [22]. It should be noted that the effect of those SNPs on *FTO* itself is still unknown. Thus, the phenotypes of *Fto* knockout mice do not necessarily contradict with the results from the human studies.

The SNPs in *FTO* that are associated with obesity in humans reside in the intronic region and their effect on the function of *FTO* remains elusive. The limited expression studies in humans found no clear association between obesity-related SNPs and the *FTO* mRNA expression levels in adipose tissue and skeletal muscle [41]–[43]. In fact, both negative and positive correlations have been observed [41], [43], complicating the effort in deciphering the effect of these SNPs on *FTO* expression. Recently, heterozygous loss-of-function *FTO* mutations have been identified in both lean and obese humans [37]. Moreover, patients carrying homozygous loss-of-function mutations in *FTO* show severe growth retardation and multiple malformations, but no record of obesity [44]. Thus, the growth retardation phenotype is shared between human and mouse when *FTO* is not functional, indicating a primary function of *FTO* in the regulation of linear growth. However, whether *FTO* plays a role in obesity needs further investigation.

The reduced serum IGF-1 levels and a significant decrease in bone mineral density in both whole body and neural *Fto* knockout models argue for a function of *Fto* in the hypothalamus-pituitary axis. This axis controls the expression and secretion of IGF-1 by the liver through the action of growth hormone (GH), and produces a host of other endocrine factors involved in the regulation of many aspects of normal physiology including mineral metabolism in the bone [45], [46]. GH deficiency is associated with obesity in humans [47] and the loss of GH function due to a missense mutation in mice results in disproportional increases in body fat despite an overall reduction in body size and weight [48]. It is possible that the *Fto* mutant mice suffer some degrees of GH deficiency. Determining whether or not FTO regulates GH and/or other hormones secreted by the hypothalamus-pituitary axis will greatly facilitate the elucidation of FTO's physiological function in future.

Our neural-specific knockout mice recapitulated essentially all of the phenotypes in the complete knockout mice. This indicates that FTO function in the brain is crucial in spite of its ubiquitous expression. The analysis of mice with sub-regional deletion of *Fto* in the brain, especially, the hypothalamus-specific deletion, will pinpoint where exactly *Fto* functions.

As a member of the Fe (II) and 2-oxoglutarate (2-OG)-dependent oxygenase superfamily [25]–[27], FTO belongs to the AlkB subfamily [25]–[27], [30]. AlkB proteins are important enzymes catalyzing the removal of alkyl adducts from DNA in both prokaryote and eukaryote organisms [49]. More recent biochemical analysis suggested that FTO might be a RNA demethylase [30], and recent structure analysis of FTO also supported its preference for single-stranded over double-stranded nucleic acids [25]. This is more consistent with a role of FTO in regualting gene expression (at the posttranscriptional level) than in reparing DNA. Likewise, a role of FTO in the regulation of gene expression is more fitting with the phenotypes of *Fto* mutnat mice than a role in DNA repair. Knowing where exactly FTO funcitons will assist greatly in the identification of its physiological substrates. The conditonal *Fto* knockout mice we have generated will be helpful in that regard.

## Materials and Methods

### Ethics Statement

All animal experiments were performed according to the protocol approved by the Institutional Animal Care and Use Committee (IACUC) of Baylor College of Medicine. The Protocol number is AN-5002.

### Western blot analysis and antibodies

For Western blot analysis, tissues were lysed in RIPA buffer and the protein concentration was determined with Bradford method. We raised rabbit anti-mFTO antisera and affinity-purified the antibodies for the use in immunoblotting. Anti-α-TUBULIN antibodies were obtained from Sigma (T 5168).

### Generation of *Fto* conditional knockout mice

The genomic DNA of targeting mouse *Fto* sequence containing exon 3 (about 9 kb) was isolated from a BAC clone (Sanger Center, UK) by gap repair. The construction of the conditional targeting vector was carried out via homologous recombination in E. coli [50]. The vector was linearized and introduced into E14Tg2a.4 ES cells. Recombinant ES clones were identified by Southern blot analysis and used to produce chimeric mice.

The F1 *Fto* conditional knockout mice (*Fto^+/cko^*) were bred with *Meox2-Cre* mice (JAX 003755) expressing Cre recombinase in all the epiblast-derived tissues to delete exon3. The progenies were backcrossed to C57BL/6 mice for at least two more generations. The heterozygotes (F3 and beyond) were then intercrossed to generate complete knockout mice.

F1 *Fto^+/cko^* mice were also bred with mice expressing *FLP1* recombinase (JAX 003946) to first delete the selection cassette flanked by the Frt sites. The mice with one floxed *Fto* allele (*Fto^+/flox^*) were then bred with *Meox2-Cre* mice (JAX 003755) to delete exon 3. Similarly, the progenies with one *Fto* knockout allele (having no selection cassette now) were backcrossed to C57BL/6 mice for at least three more generations. The heterozygotes were then intercrossed to generate knockout mice.

To generate brain specific *Fto* deletion, *Fto^flox/flox^* mice were crossed with *Nestin-Cre* mice (JAX 003771) to generate *Fto^+/flox^*/*Tg(Nes-Cre)* mice, which were then crossed to *Fto^flox/flox^* mice to generate *Fto^N+^* (*Fto^flox/flox^* and *Fto^+/flox^*, for control) and *Fto^NΔ^* (*Fto^flox/flox^/Nes-Cre*) mice.

### Animal experiments

Animals were housed in a specific pathogen free facility at 22±2°C under a cycle of 12 hr light (7:00 am light on) and 12 hr dark (7:00 pm light off). They have free access to water and food (normal chow: 2920X Teklad Rodent Diet). For 24 hr fasting experiments, the food was removed from the cages at 6:00 pm, but the mice had free access to water. For high fat diet challenge, normal chow was substituted by 60 kcal% fat diet (Research Diets, Inc. D12492).

Body composition and bone mineral density were measured with Lunar PIXImus dual energy x-ray absorptiometry (DEXA) densitometry following the standard protocol.

### Measurement of the rates of metabolism and food consumption

The metabolic rates of mice were measured by indirect caloriometry using the Comprehensive Lab Animal Monitoring System (CLAMS, Columbus Instruments). The food intake was also monitored by the system. 16∼17-week-old mice were housed individually in the monitoring cage with free access to water and food for 3 days for acclimatization. Then the lean mass of the mice was measured by MRI (EchoMRI), before the animals were put back into the monitoring cages and monitored for the next 3 days for oxygen consumption, carbon dioxide production and food intake.

### IGF-1 ELISA

Blood was collected from 4-week-old mice by cardiac puncturing. The sera were aliquoted and snap-frozen in liquid nitrogen. Before ELISA, the sera were extracted by acid-ethanol with cryo-precipitation to release IGF-1 from its binding proteins [51]. IGF-1 ELISA was performed using commercial kits (Signosis EA-2204, and AssayPro EMI1001-1) according to the manufacturers' protocols. The results were presented as the read-out from the microplate reader (spectrometer).

### Statistic analysis

All statistic analyses were performed by one-way ANOVA (three sample sets) or unpaired t-test (two sample sets). ANCOVA was used when indicated.

## Supporting Information

Figure S1 Generation and characterization of *Fto* gene-trap mice. (A) Schematic representation of *Fto* gene-trap strategy. (B) Schematic representation of predicted *Fto* wildtype and gene-trap coding sequence (CDS). (C) Western blot analysis of tissues from mice of all genotypes. (D) Growth curves of *Fto* ^+/+^ and *Fto* ^gt/gt^ mice. For each genotype (*Fto* ^+/+^/*Fto* ^gt/gt^), n = 16/15 (males); 12/16 (females). All values are mean ± s.e.m. (E) The body length of adult *Fto* ^+/+^ and *Fto* ^gt/gt^ mice. At the time of the measurement, males were 13∼14.5-week-old, and females 13∼16-week-old. For each genotype (*Fto* ^+/+^/*Fto* ^gt/gt^,), n = 10/9 (males); 7/7 (females). All values are mean ± s.e.m. (F) Body composition (fat mass/total tissue mass %) of *Fto* ^+/+^ and *Fto* ^gt/gt^ mice fed on normal chow or high fat diet. Body composition was measured by DEXA (dual energy X-ray absorptiometry). For normal chow group, males were 13∼14.5-week-old, and females 13∼16-week-old. For each genotype (*Fto* ^+/+^/*Fto* ^gt/gt^,), n = 10/9 (males); 7/7 (females). For the high fat diet group, at the time of measurement, the mice had been fed on high fat diet (60 kcal % fat) for 12 weeks from 6-week-old. For each genotype (*Fto* ^+/+^/*Fto* ^gt/gt^,), n = 11/6 (males); 8/8 (females). Statistical analyses were performed by unpaired t-test. All values are mean ± s.e.m.
(0.74 MB TIF)

Figure S2 Dermatitis in *Fto* knockout mice after high fat diet regimen. (A) A representative picture of an *Fto^Δ^* ^/*Δ*^ mice suffering dermatitis around the neck area. (B) A representative picture of *Fto* ^+/+^ and *Fto^Δ^* ^/*Δ*^ mice after the high fat diet. The asterisk denotes the one with dermatitis.
(0.74 MB TIF)

Figure S3 Metabolic rate of *Fto* mutant mice analyzed by ANCOVA. (A, C) Average hourly O_2_ consumption (A) and average hourly CO2 production (C) in relation to lean mass of 16∼17-week-old male *Fto* ^+/*Δ*^ and *Fto^Δ^* ^/*Δ*^ mice during light and dark period. (B, D) Average hourly O_2_ consumption (B) and average hourly CO_2_ production (D) of 16∼17-week-old male *Fto* ^+/*Δ*^ and *Fto^Δ^* ^/*Δ*^ mice adjusted by ANCOVA using an average lean mass. In (A)–(D), n = 7/7 (*Fto* ^+/*Δ*^/*Fto^Δ^* ^/*Δ*^). In (B) and (D), statistical analyses were performed by unpaired t-test using adjusted data. **P<0.01. All values are mean ± s.e.m. (E, F) Average hourly O_2_ consumption (E) and CO_2_ production (F) in relation to lean mass of 16∼17-week-old male *Fto^N^* ^+^ and *Fto^NΔ^* mice during light and dark period. n = 7/9 (*Fto^N^* ^+^/*Fto^NΔ^*). No significant linear relationship was detected.
(0.50 MB TIF)

Table S1 Genotypes of 10∼14-day-old pups from heterozygote intercrosses.
(0.04 MB PDF)

Table S2 Genotypes of 1∼3-day-old pups from heterozygote intercrosses that were found dead or missing.
(0.03 MB PDF)

Table S3 Genotypes of E14.5∼18.5 embryos from heterozygote intercrosses.
(0.03 MB PDF)

Table S4 Number of mice that developed dermatitis on high fat diet.
(0.04 MB PDF)
