# Supplementary figures and images for: The Fat Mass and Obesity Associated Gene FTO Functions in the Brain to Regulate Postnatal Growth in Mice
Source: PLoS One. 2010 Nov 16;5(11):e14005. doi: 10.1371/journal.pone.0014005 (PMC2982835; doi:10.1371/journal.pone.0014005)

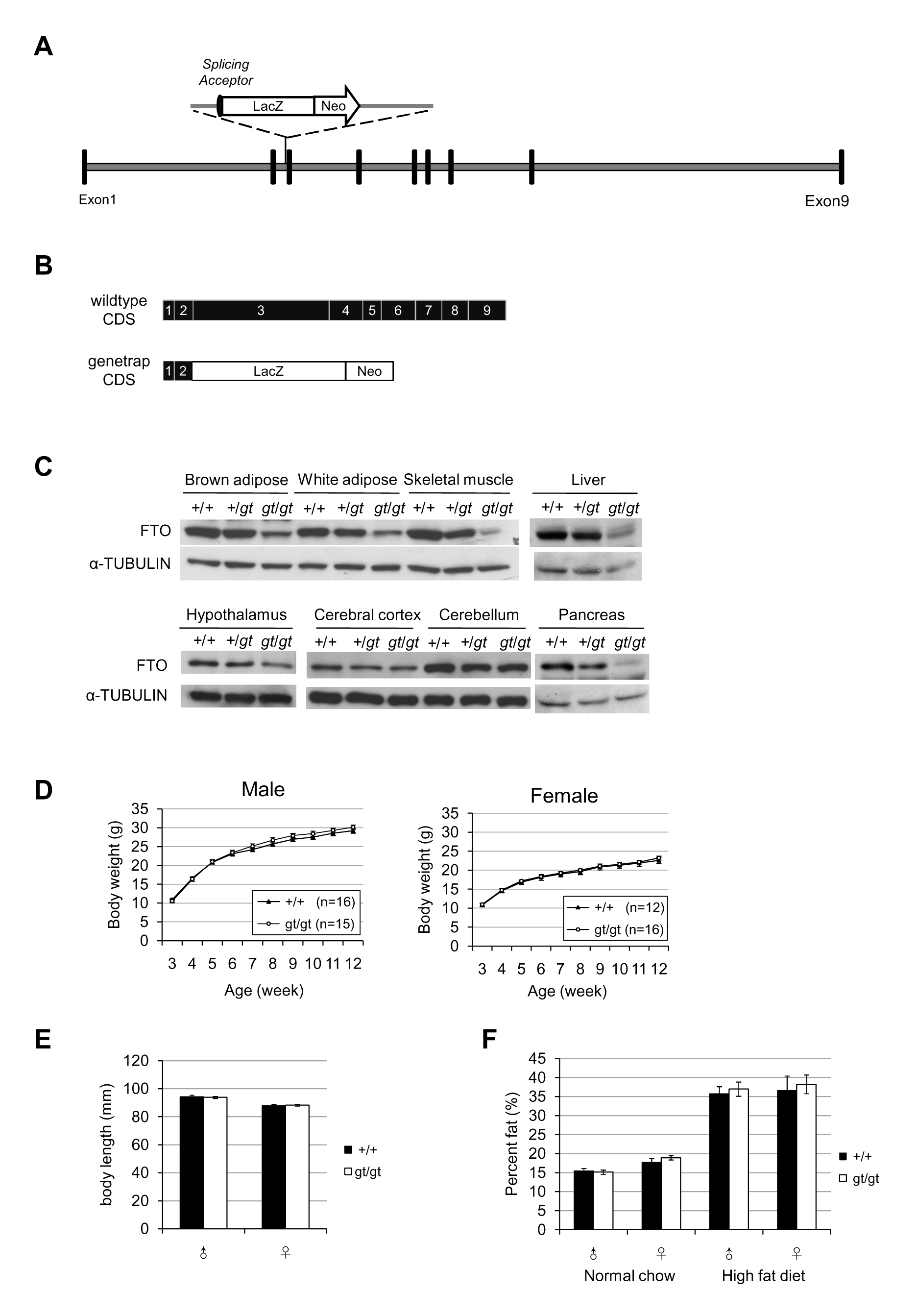

Supplement: Figure S1 — Generation and characterization of Fto gene-trap mice. (A) Schematic representation of Fto gene-trap strategy. (B) Schematic representation of predicted Fto wildtype and gene-trap coding sequence (CDS). (C) Western blot analysis of tissues from mice of all genotypes. (D) Growth curves of Fto +/+ and Fto gt/gt mice. For each genotype (Fto +/+/Fto gt/gt), n = 16/15 (males); 12/16 (females). All values are mean ± s.e.m. (E) The body length of adult Fto +/+ and Fto gt/gt mice. At the time of the measurement, males were 13∼14.5-week-old, and females 13∼16-week-old. For each genotype (Fto +/+/Fto gt/gt,), n = 10/9 (males); 7/7 (females). All values are mean ± s.e.m. (F) Body composition (fat mass/total tissue mass %) of Fto +/+ and Fto gt/gt mice fed on normal chow or high fat diet. Body composition was measured by DEXA (dual energy X-ray absorptiometry). For normal chow group, males were 13∼14.5-week-old, and females 13∼16-week-old. For each genotype (Fto +/+/Fto gt/gt,), n = 10/9 (males); 7/7 (females). For the high fat diet group, at the time of measurement, the mice had been fed on high fat diet (60 kcal % fat) for 12 weeks from 6-week-old. For each genotype (Fto +/+/Fto gt/gt,), n = 11/6 (males); 8/8 (females). Statistical analyses were performed by unpaired t-test. All values are mean ± s.e.m. (0.74 MB TIF) [file pone.0014005.s001.tif]

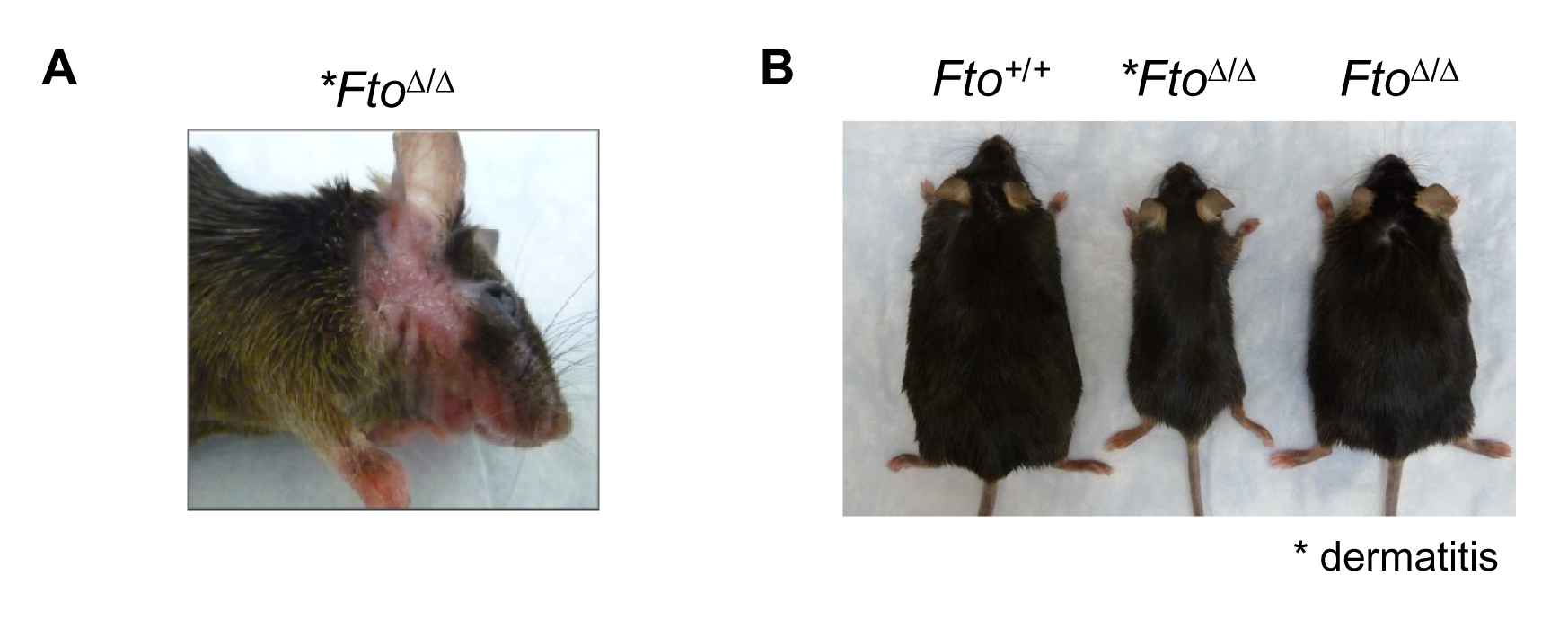

Supplement: Figure S2 — Dermatitis in Fto knockout mice after high fat diet regimen. (A) A representative picture of an FtoΔ /Δ mice suffering dermatitis around the neck area. (B) A representative picture of Fto +/+ and FtoΔ /Δ mice after the high fat diet. The asterisk denotes the one with dermatitis. (0.74 MB TIF) [file pone.0014005.s002.tif]

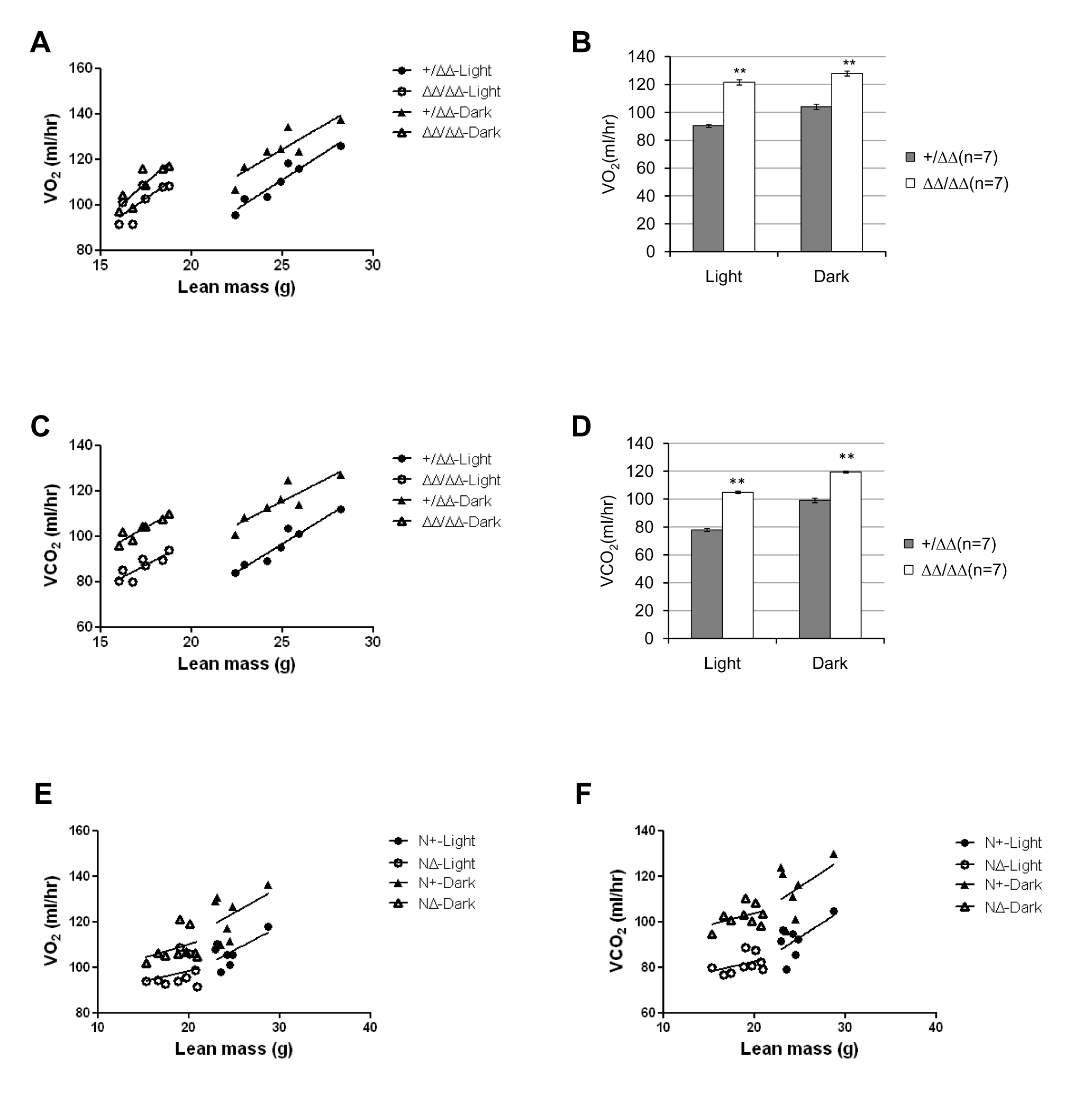

Supplement: Figure S3 — Metabolic rate of Fto mutant mice analyzed by ANCOVA. (A, C) Average hourly O2 consumption (A) and average hourly CO2 production (C) in relation to lean mass of 16∼17-week-old male Fto +/Δ and FtoΔ /Δ mice during light and dark period. (B, D) Average hourly O2 consumption (B) and average hourly CO2 production (D) of 16∼17-week-old male Fto +/Δ and FtoΔ /Δ mice adjusted by ANCOVA using an average lean mass. In (A)–(D), n = 7/7 (Fto +/Δ/FtoΔ /Δ). In (B) and (D), statistical analyses were performed by unpaired t-test using adjusted data. **P<0.01. All values are mean ± s.e.m. (E, F) Average hourly O2 consumption (E) and CO2 production (F) in relation to lean mass of 16∼17-week-old male FtoN + and FtoNΔ mice during light and dark period. n = 7/9 (FtoN +/FtoNΔ). No significant linear relationship was detected. (0.50 MB TIF) [file pone.0014005.s003.tif]
