# Supplementary material for: The Fat Mass and Obesity Associated Gene FTO Functions in the Brain to Regulate Postnatal Growth in Mice
Source: PLoS One. 2010 Nov 16;5(11):e14005. doi: 10.1371/journal.pone.0014005 (PMC2982835; doi:10.1371/journal.pone.0014005)
Supplement: Table S1 — Genotypes of 10∼14-day-old pups from heterozygote intercrosses. (0.04 MB PDF) [file pone.0014005.s004.pdf]

Table S1

Genotypes of 10~14-day-old pups from heterozygote intercrosses

	+/+	+/Δ	Δ/Δ
♀	262(29.5%)	510 (57.4%)	117 (13.2%)
♂	216 (27.4%)	454 (57.5%)	119 (15.1%)
total	478 (28.5%)	964 (57.4%)	236 (14.1%)
