# Supplementary material for: The Fat Mass and Obesity Associated Gene FTO Functions in the Brain to Regulate Postnatal Growth in Mice
Source: PLoS One. 2010 Nov 16;5(11):e14005. doi: 10.1371/journal.pone.0014005 (PMC2982835; doi:10.1371/journal.pone.0014005)
Supplement: Table S2 — Genotypes of 1∼3-day-old pups from heterozygote intercrosses that were found dead or missing. (0.03 MB PDF) [file pone.0014005.s005.pdf]

Table S2

Genotypes of 1~3-day-old pups from heterozygote intercrosses that were found dead or missing

+/+	+/Δ	Δ/Δ	unknown genotype
5	14	43	74
