# Supplementary material for: The Fat Mass and Obesity Associated Gene FTO Functions in the Brain to Regulate Postnatal Growth in Mice
Source: PLoS One. 2010 Nov 16;5(11):e14005. doi: 10.1371/journal.pone.0014005 (PMC2982835; doi:10.1371/journal.pone.0014005)
Supplement: Table S3 — Genotypes of E14.5∼18.5 embryos from heterozygote intercrosses. (0.03 MB PDF) [file pone.0014005.s006.pdf]

Table S3

Genotypes of E14.5~18.5 embryos from heterozygote intercrosses

	+/+	+/Δ	Δ/Δ
total	15 (23.4%)	31 (48.4%)	18 (28.1%)
