# Supplementary material for: The Fat Mass and Obesity Associated Gene FTO Functions in the Brain to Regulate Postnatal Growth in Mice
Source: PLoS One. 2010 Nov 16;5(11):e14005. doi: 10.1371/journal.pone.0014005 (PMC2982835; doi:10.1371/journal.pone.0014005)
Supplement: Table S4 — Number of mice that developed dermatitis on high fat diet. (0.04 MB PDF) [file pone.0014005.s007.pdf]

Table S4

Number of mice that developed dermatitis on high fat diet

	Fto$^{+/+}$	*Fto*$^{+/\Delta}$	*Fto*$^{\Delta/\Delta}$
Cases of dermatitis / total number	0/16 (male)<br>0/12 (female)	0/14 (male)<br>0/9 (female)	9/19 (male)<br>11/17 (female)
